# Elucidating Scarab Divergence in an Evolutionary-Ecological Context through the Comprehensive Analysis of the Complete Mitogenome of *Anomala*

**DOI:** 10.3390/genes15081022

**Published:** 2024-08-03

**Authors:** Xianyi Wang, Shuchai Li, Tielong Xu

**Affiliations:** 1 Engineering Research Center of Medical Biotechnology, School of Biology and Engineering, Guizhou Medical University, Guiyang 561099, China; wangxianyi@gmc.edu.cn; 2 Guizhou Provincial Engineering Research Center of Medical Resourceful Healthcare Products, Guiyang Healthcare Vocational University, Guiyang 550081, China; 18984851728@163.com

**Keywords:** mitogenome, *Anomala*, phylogenetic, diet

## Abstract

*Anomala* Samouelle, 1819 is one of the specious genera of Coleoptera, with over 1000 known species, and includes some of the most destructive pests of crops or forests. Morphological convergence is a common phenomenon within this genus, making the identification of closely related species very difficult. To explore the phylogenetic placement of Anomalini and provide a basis for the classification and identification of *Anomala*, we comparatively analyzed the complete mitogenome of three *Anomala* species (*A. exoleta*, *A. perplexa* diana, and *A. praecoxalis*). Based on all accessible mitogenome data, we performed comparative mitochondrial genomics analysis of this genus and reconstructed the phylogenetic relationships of Scarabaeidae based on two datasets (protein-coding genes and amino acids) and two methods (Bayesian approach and maximum likelihood). The phylogenetic relationships found in this study highly support that the groups of Aphodiinae, Cetoniinae, Dynastinae, Rutelinae and Scarabaeinae are monophyletic. Interestingly, the phylogenetic clustering relationship was highly consistent with the Scarabaeidae diet, indicating that the herbivorous species and dung-eating species are clustered separately. The phylogenetic tree showed that the subfamily Melolonthinae and the genus *Anomala* are not monophyletic, suggesting that these two groups should be further revised with more data.

## 1. Introduction

Coleoptera is one of the most biodiversity-rich groups within insecta. Rutelinae, a subfamily of the family Scarabaeidae (Coleoptera, Polyphaga), is mainly distributed in the Indomalayan, Palaearctic, and Afrotropical regions. In addition, the biodiversity of Rutelinae is extremely rich, with over 4200 recorded species in 235 genera from seven tribes worldwide. In China alone, three tribes, 25 genera, and 526 species have been reported. Rutelinae beetles are voracious feeders, capable of consuming large amounts of food in a short period. They pose significant ecological and economic threats through their prolonged harmful activity and wide distribution. Adults of the family Rutelinae harm the buds, leaves, flowers, and fruits of plants [1]. Their larvae are significant underground pests, causing harm to trees, lawns, crops, and other plant roots and adversely affecting the ecology and cash crop. Rutelinae were considered a distinct subfamily based on a few morphological features. The dominant distinguishing feature between scarab beetles and other subfamilies of the family scarab is moveable and asymmetrical claws, which are usually split [2]. Based on current morphological and molecular evidence, it is believed that the Rutelinae and Dynastinae form a common lineage; however, their monophyletism needs to be verified. Howden (1982) reconstructed a phylogenetic relationship of Scarabaeoidea based on the morphological characteristics (the base of antennae had well-developed setae, the base of claws had no teeth, the size of claws varied, the abdominal valve [3,4,5,6,7] nodes were nearly parallel, etc.) and found that Rutelinae and Dynastinae gather in a cluster [3]. Browne and Scholtz (1998) showed that Rutelinae and Dynastinae shared five common characteristics based on the morphology of the wing bases; however, they could not distinguish the two subfamilies by the wing bases [4]. Multiple studies based on molecular evidence have yielded similar results [5,6]. However, the phylogenetic relationship between Rutelinae and Dynastinae is still not well explained, and there is a lack of systematic research on the Rutelinae species.

With the development of molecular sequencing technology and the low cost of sequencing, DNA taxonomy has become one of the most promising methods to solve the taxonomic problems of this subfamily. For Rutelinae, molecular phylogenetics studies used to rely on mitochondrial single genes, nuclear gene fragments, or combinations of two or three genes. Mitochondrial genes such as *cox1*, *cox2*, *16S*, *12S*, *cytb* and *nad1* are molecular markers frequently used in evolutionary analysis [7]. However, a single gene not only contains less evolutionary information, which makes it difficult to completely analyze the phylogenetic relationships among species, but also exhibits differences in selection pressure and evolutionary rate among different genes, which may lead to “long branch attraction” and other issues. Mitochondria are semi-autonomous organelles that exist in eukaryotic cells. The mitochondrial genome serves as a molecular sequence that can autonomously perform genetic functions. It is widely used in species identification, population genetics, biogeography, and phylogeny [8,9].

*Anomala* is the largest genus of the Rutelinae, with over 1300 species worldwide, covering two-thirds of the species in the tribe Anomalini. *Anomala* also has the most taxonomic problems, especially in terms of identifying closely related species. To date, only 10 mitochondrial genomes from Rutelinae have been recorded in NCBI (of which Anomala has only three mitochondrial genome sequences). In this study, we sequenced, assembled, and annotated the mitochondrial genomes of three taxa of *Anomala*, namely *A. exolete*, *A. perplexa* diana, and *A. praecoxalis*. A comparative analysis of mitochondrial genomes was then conducted, and a phylogenetic tree of Scarabaeidae based on a Bayesian approach (BI) and method of maximum likelihood (ML) was constructed to analyze phylogenetic relationships. This approach primarily solves the following scientific problems: (1) determining the status of Rutelinae in the Scarabaeidae classification; (2) clarifying the basic characteristics of the mitochondrial genome of *Anomala*; and (3) assessing whether the monophyletic group of *Anomala* is formed.

## 2. Materials and Methods

### 2.1. Specimen Collection and DNA Extraction

*A. exoleta* specimens were collected from Xigu district, Lanzhou City, Gansu Province, China, on 28 June 2020; *A. perplexa* diana were collected from Mêdog County, Tibet, China, on 18 August 2020; and *A. praecoxalis* were collected from Dawei Mountain National Nature Reserve, Yunnan Province, China, on 2 May 2020, and instantly stored in anhydrous ethanol. The samples were transported to the laboratory for immediate storage at −20 °C until characterization or DNA extraction. These three species were accurately identified based on morphological characteristics. The extraction of DNA samples for sequencing was conducted using a DNeasy© Tissue Kit as per the specification manual of the manufacturer.

### 2.2. Mitogenome Sequencing, Assembly, and Annotation

The mitogenomes for three *Anomala* species were sequenced by Berry Genomics on a HiSeq 2500 platform (Illumina) with 150 bp paired-end reads. The average insert size was 350 bp, and 6 GB of clean data was obtained. The mitogenomes were assembled using Geneious Prime 2020.2.1 software [10] and were based on a mitochondrial reference sequence of *A. rufiventris* (OR208200). The assembled mitogenome sequence was compared with the homologous sequences of *A. corpulenta* NC069575, *A. rufiventris* OR208200, and *A. russiventris* NC065310 [11], which were obtained from GenBank, and the accuracy of the sequences was confirmed by performing a BLAST search on NCBI [12]. The assembled mitogenome was annotated utilizing the MITOS network services and the invertebrate mitochondrial hereditary code [13]. The placement of 22 tRNAs was controlled utilizing tRNAscan-SE version 1.21 and ARWEN version 1.2 [14,15]. Two rRNA genes were confirmed based on the position of proximity tRNA genes and were defined as the other *Anomala* whose mitogenome sequences were previously published in GenBank. The circular mitogenomic map was created using OGDraw version 1.3.1 [16].

### 2.3. Sequence Analysis of Mitogenomes

Base composition of *Anomala* mitogenomes was assessed using DNASTAR Lasergene v7.1 (http://www.dnastar.com (accessed on 28 June Month 2024)). Strand asymmetries in Rutelinae mitogenomes were calculated using the following formulas: GC skew = [G − C]/[G + C] and AT skew = [A − T]/[A + T]. Codon usage patterns within the *Anomala* mitogenome were analyzed using Sequence Manipulation Suite based on genetic code 5.

### 2.4. Sequence Alignments and Phylogenetic Analyses

The Scarabaeidae of 30 representative species (all Rutelinae genomes available and one random species per other genera) were selected for systematic evolution as the ingroup, and two species, *Sinodendron yunnanense* (NC036157) and *Pseudorhaetus sinicus* (NC069553), were chosen as outgroups (Table 1) [11,17,18,19,20,21,22,23,24,25]. The two datasets (protein-coding genes [PCG]: 13; and amino acids [AA]: amino acid sequences of 13 PCGs) were used to analyze the phylogenetic relationships within Scarabaeidae. Initially, the alignment of each PCG was conducted utilizing the MAFFT arithmetic in the Translator X server [26]. Ultimately, all sequences were evaluated and hand-corrected with MEGA7 [27]. To test whether the two datasets were suited for high-level phylogenetic inferences, the heterogeneity in nucleotide divergence was evaluated via pairwise comparisons in a multiple sequence alignment using AliGROOVE v1. 05 [28]. Based on two different datasets containing 32 species, we used ML and BI methods to reconstruct phylogenetic relationships. An ML phylogenetic tree was reconstructed using IQ-TREE with 10,000 iterations via a super-rapid bootstrap approximation [29]. BI system phylogeny was executed by MrBayes 3.3 [30]. The BI analysis used the default settings and simulated four standalone runs for an aggregate of 0.1 billion generations, sampling once per 1000 generations. When the convergence value was less than 0.01, the operation was stopped. The tree was stored every 1000 generations, and the first 25% was discarded based on the burn-in parameter. Finally, a 50% merge tree was formed. The phylogenetic trees were visualized using FigTree 1.4.4 (http://tree.bio.ed.ac.uk/software/figtree/) and beautified using Adobe Illustrator CC 22. 1. Software.

## 3. Results

### 3.1. Mitogenomic Characteristics of Anomala Species

**Figure 1:**
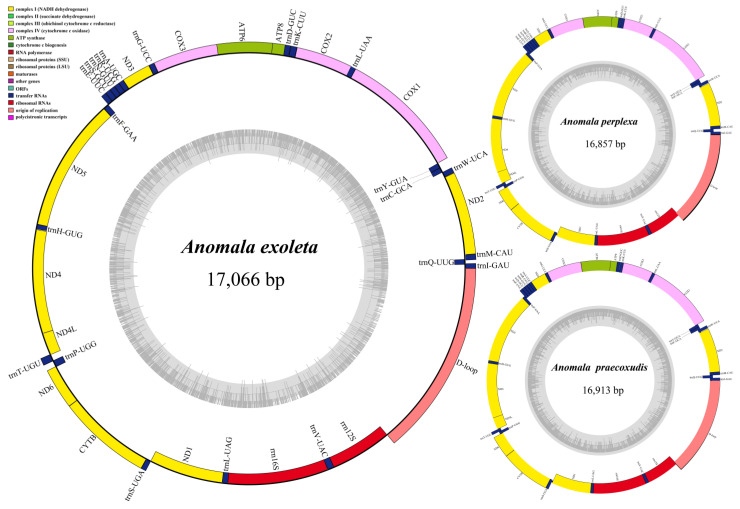
Circular maps of the mitogenomes of three newly sequenced *Anomala* species.

This study reports the complete mitochondrial genome sequences of three *Anomala* species: *A. exolete*, *A. perplexa* diana, and *A. praecoxalis*, which were sequenced and found to be 17,066 bp, 16,857 bp, and 16,913 bp in length, respectively. Comparative genomic analysis of these mitochondrial genomes with those of *A. corpulenta* (NC069575), *A. rufiventris* (OR208200), and *A. russiventris* (NC065310) obtained from NCBI revealed high conservation in gene content and order. Mitochondrial genome length in the studied Rutelinae species varied from 15,601 bp (*A. russiventris*) to 17,240 bp (*A. rufiventris*). Comparative analysis with *Drosophila yakuba* revealed conserved gene order and orientation, with no evidence of gene rearrangements in any *Anomala* species. The mitochondrial genome of all six *Anomala* species encodes 37 genes, including 13 PCGs, 22 transfer ribonucleic acid (tRNA) genes, and two ribosomal ribonucleic acid (rRNA) genes. Nine PCGs and 14 tRNA genes are encoded on the J-strand, whereas the remaining four PCGs, eight tRNA genes, and two rRNA genes are encoded on the N-strand (Figure 1, Table 2).

In the sequenced mitochondrial genome of Rutelinae insects, ATT, TTA, TTT, and ATA were the main codons used, whereas GC-rich codons (such as GCG, CGG, and CTC) were used less frequently. Most PCGs start their transcription with ATN. *Anomala* insect gene transcription is often terminated using TAA, TAG, or an incomplete codon T- (Table 2).

### 3.2. Codon Usage Analysis

**Figure 2:**
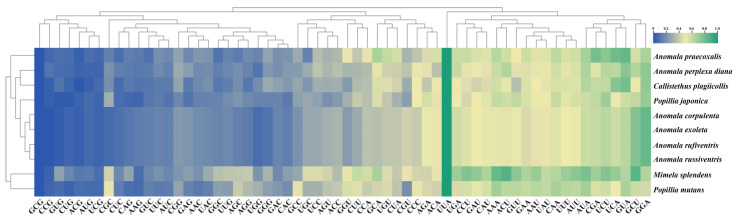
RSCU heatmap of 10 Rutelinae mitogenomes.

Energy expenditure and translation rate are directly affected by codon usage. Mitochondrial genes prefer specific codons that help optimize the efficiency of translation and conserve energy for cellular functions. We compared 10 mitogenomes to analyze the codon usage trends in different Rutelinae mitogenomes (Figure 2). This set comprised seven mitogenomes that had already been published in the database, along with three *Anomala* mitogenomes that we obtained in the current study. Our extensive analysis showed that the most common codon in the mitogenomes of 10 Rutelinae species was UUA (representing leucine; Leu). Rutelinae mitogenomes predominantly initiated translation of PCGs using the start codon ATG, whereas the most frequent stop codon was TAA, followed by TAG.

**Figure 3:**
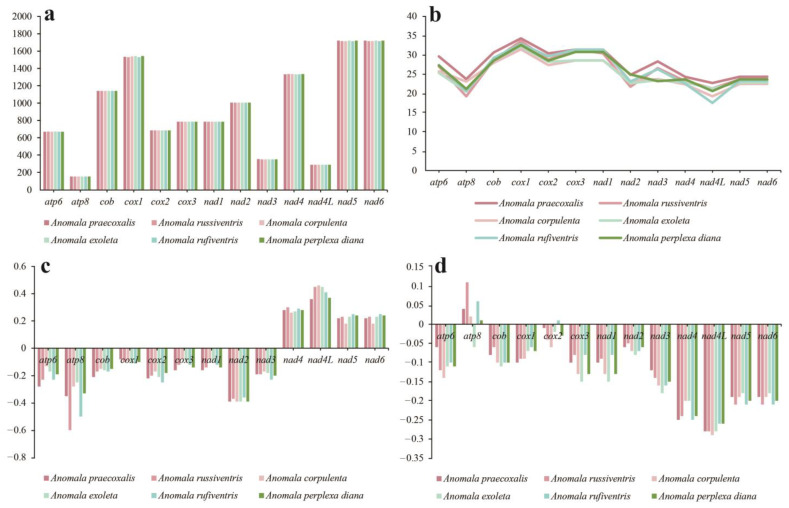
Variation in length and base composition of the 13 protein-coding genes (PCGs) among five *Anomala* mitochondrial genomes. (**a**) PCG length variation; (**b**) GC content of PCGs; (**c**) AT skew; (**d**) GC skew.

*Anomala*’s 13 PCGs are extremely conserved in length, except for *cox1*, *nad5*, and *nad6*, with the others being approximately the same length (Figure 3a). The GC content of these 13 PCGs in the mitochondrial genomes of the six *Anomala* species was greatest for *cox1*, while *atp8* and *nad4l* had the lowest GC content relative to other PCGs (Figure 3b). Except for *nad4*, *nad4L*, *nad5*, and *nad6*, all other PCGs had negative values of AT skew. Except for *atp6* and *atp8*, which showed differences of AT skew between different species, other PCGs had similar AT skew values (Figure 3c). The GC skew of *atp8* was found to be positive in five species (*A. praecoxalis*, *A. russiventris*, *A. corpulenta*, *A. exoleta*, and *A. perplexa diana*), whereas the GC skew of all genes in other species was negative (Figure 3d).

### 3.3. Phylogenetic Relationships

**Figure 4:**
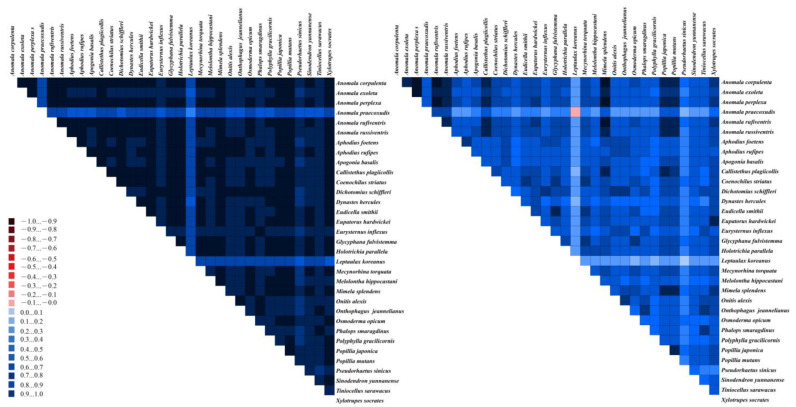
Heterogeneity of AA (**left**) and PCG (**right**) datasets in Scarabaeidae mitogenomes. Color gradients from dark red (−1) to dark blue (+1) represent levels of heterogeneity between sequences, with darker shades indicating greater heterogeneity.

Based on the calculation results obtained from the AliGROOVE 1.06 software, the heterogeneity of PCG and AA datasets in the mitogenomic data of Scarabaeidae was found to be weak (Figure 4). Hence, the two datasets could be used to reconstruct a phylogenetic tree.

**Figure 5:**
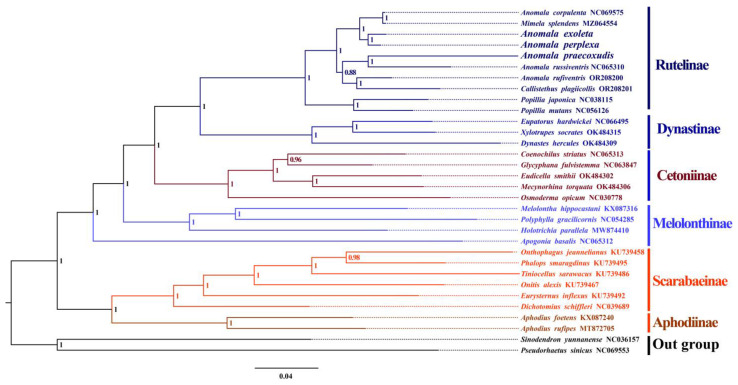
Phylogenetic trees of Scarabaeidae inferred by the MrBayes 3.2.6 method-based amino acid sequences of PCGs.

In this study, we used two methods (BI and ML) to construct phylogenetic trees from two datasets (PCG and AA). The support rate of the BI-based method was higher than that of the ML-based method. Phylogenetic trees reconstructed using these methods were highly consistent across different datasets. In all phylogenetic relationships, the monophyletic nature of all Scarabaeidae subfamilies, except for Melolonthinae, was well supported (BP, bootstrap percentage = 100, PP, Posterior Probability = 1). Specifically, Scarabaeinae and Aphodiinae, as well as Rutelinae and Cetoniinae, formed sister groups. Within Rutelinae, the monophyly of *Popillia* was well verified (BP = 100, PP = 1). However, the monophyly of *Anomala* was questioned, as it formed different branches from *M*. *splendens* and *C*. *plagiicollis* (Figure 5 and Figures S1–S3).

## 4. Discussion

In this study, we obtained complete mitochondrial genomes of three species (*A. exolete*, *A. perplexa diana*, and *A. praecoxalis*) from subfamily Rutelinae of the Scarabaeidae. We then performed comparative mitochondrial genomics analysis of this genus based on two datasets (PCG and AA) and two methods (Bayesian approach and method of ML), and the phylogenetic relationships of Scarabaeidae were reconstructed. The high support of this study indicated that the groups of Aphodiinae, Cetoniinae, Dynastinae, Rutelinae, and Scarabaeinae were monophyletic. Most genera of these subfamilies are monophyletic groups, which is consistent with previous studies [17]. The systematic relationships constructed in this study show that scarab insects with the same diet congregate, that is, the dung-eating Scarabaeinae and Aphodiinae cluster into a branch, whereas the herbivorous subfamilies Cetoniinae, Dynastinae, Melolonthinae, and Rutelinae form another cluster, which is consistent with previous studies [16,19]. This study supports the idea that fecal-eating behavior predates herbivorous eating, which aligns with previous findings by Ayivi et al. and Song and Zhang [16,19]. The two subfamilies of Dynastinae and Rutelinae are sister groups of each other, which is consistent with the prevailing view based on morphological and molecular evidence [30]. Simultaneously, our phylogenetic relationship analysis indicates that the subfamily Melolonthinae is not monophyletic, with *Apogonia* being isolated from other Melolonthinae, which is consistent with previous reports [5,17,19,20,31,32].

In addition, the phylogenetic tree showed that the *Anomala* species clustered into three branches. In the first branch, *A. corpulenta* and *M*. *splendens* formed sister species, whereas *A. perplexa* diana and *A. exoleta* formed another sister group. The second branch was formed by *A. praecoxalis* and *A. russiventris*, whereas the third branch was formed by *A. rufiventris* and *C*. *plagiicollis*. The second and third branches are sister groups to each other and, together, sister to the first branch. The relatively short branch length between *M*. *splendens* (MZ064554) and *A. corpulenta* (NC069575), despite their morphological divergence, is unusual. Therefore, we propose that *Anomala* is not monophyletic and recommend further revision of this genus with additional taxa in future studies.

## 5. Conclusions

In this study, we obtained complete mitochondrial genome sequences of three species in *Anomala* (*A. exoleta*, 17,066 bp in size; *A. perplexa* diana, 16,857 bp in size; and *A. praecoxalis*, 16,913 bp in size). We performed a comparative genomic analysis of *Anomala* mitochondrial genomes with those of the existing genus *Anomala* genus on NCBI. The results showed that mitochondrial genomes across all *Anomala* species are highly conserved in length and gene arrangement. Phylogenetic relationships were then constructed based on two datasets (PCG and AA) and two methods (Bayesian and ML). Our study indicates that Aphodiinae, Cetoniinae, Dynastinae, Rutelinae, and Scarabaeinae are monophyletic groups. The analysis also shows that dung-eating scarabs (Scarabaeinae and Aphodiinae) and herbivorous scarabs (Cetoniinae, Dynastinae, Melolonthinae, and Rutelinae) cluster together. Additionally, Dynastinae and Rutelinae are sister groups. However, the phylogenetic relationships indicate that the subfamily Melolonthinae and the genus Anomala within Rutelinae are not monophyletic, suggesting the need for further revision of these groups.

## Figures and Tables

**Table 1:** Information of samples for phylogenetic analyses.

Taxa	Genus	GenBank	Size (bp)	References
Aphodiinae	*Aphodius foetens*	KX087240	15,907	Unpublished
	*Aphodius rufipes*	MT872705	15,803	Unpublished
Cetoniinae	*Coenochilus striatus*	NC065313	15,480	[11]
	*Eudicella smithii*	OK484302	16,712	[17]
	*Glycyphana fulvistemma*	NC063847	16,701	Unpublished
	*Mecynorhina torquata*	OK484306	17,192	[17]
	*Osmoderma opicum*	NC030778	15,341	[18]
Dynastinae	*Xylotrupes socrates*	OK484315	18,660	[17]
	*Eupatorus hardwickei*	NC066495	18,494	Unpublished
	*Dynastes hercules*	OK484309	17,813	[17]
Melolonthinae	*Polyphylla gracilicornis*	NC054285	16,793	Unpublished
	*Melolontha hippocastani*	KX087316	15,485	Unpublished
	*Holotrichia parallela*	MW874410	18,730	Unpublished
	*Apogonia basalis*	NC065312	15,226	[19]
Rutelinae	*A. corpulenta*	NC069575	16,673	[20]
	*A. exoleta*	PP265270	17,066	This study
	*A. perplexa diana*	PP265271	16,857	This study
	*A. praecoxalis*	PP265272	16,913	This study
	*A. rufiventris*	OR208200	17,240	Unpublished
	*A. russiventris*	NC065310	15,601	[11]
	*Callistethus plagiicollis*	OR208201	16,870	Unpublished
	*Mimela splendens*	MZ064554	15,148	Unpublished
	*Popillia japonica*	NC038115	16,541	[21]
	*Popillia mutans*	NC056126	16,192	[19]
Scarabaeinae	*Dichotomius schiffleri*	NC039689	14,802	[22]
	*Eurysternus inflexus*	KU739492	15,766	Unpublished
	*Onitis alexis*	KU739467	17,501	Unpublished
	*Onthophagus jeannelianus*	KU739458	15,654	[23]
	*Phalops smaragdinus*	KU739495	15,104	Unpublished
	*Tiniocellus sarawacus*	KU739486	15,592	Unpublished
Outgroup	*P*. *sinicus*	NC069553	18,730	[24]
	*S*. *yunnanense*	NC036157	16,921	[25]

**Table 2:** Organization of the *Anomala exolete* mitogenome.

Gene	Strand	Position	Size (bp)	Anticodon	Start Codon	Stop Codon	Intergenic Nucleotides *
*trnI*	J	1–64	64	GAT			
*trnQ*	N	62–130	69	TTG			−3
*trnM*	J	130–198	69	CAT			−1
*ND2*	J	199–1206	1008		ATG	TAA	0
*trnW*	J	1219–1286	68	TCA			12
*trnC*	N	1279–1343	63	GCA			−8
*trnY*	N	1344–1408	65	GTA			0
*COI*	J	1401–2945	1521		ATT	TAA	−8
*trnL1* (UUR)	J	2941–3005	65	TAA			−5
*COII*	J	3006–3693	651		ATC	T	0
*trnK*	J	3694–3764	71	CTT			0
*trnD*	J	3769–3833	65	GTC			4
*ATP8*	J	3834–3989	153		ATT	TAA	0
*ATP6*	J	3983–4657	666		ATG	TAA	−7
*COIII*	J	4657–5443	786		ATG	T	−1
*trnG*	J	5444–5508	65	TCC			0
*ND3*	J	5509–5862	321		ATC	TAA	0
*trnA*	J	5861–5924	64	TGC			−2
*trnR*	J	5925–5989	65	TCG			0
*trnN*	J	5990–6054	65	GTT			0
*trnS1* (AGN)	J	6055–6121	67	GCT			0
*trnE*	J	6122–6186	65	TTC			0
*trnF*	N	6185–6251	67	GAA			−2
*ND5*	N	6251–7972	1641		ATT	TAA	−1
*trnH*	N	7970–8033	64	GTG			−3
*ND4*	N	8034–9366	1332		ATA	T	0
*ND4L*	N	9363–9653	258		ATG	TAA	−4
*trnT*	J	9656–9720	65	GTG			2
*trnP*	N	9721–9785	65	TGG			0
*ND6*	J	9787–10,286	492		ATC	TAA	1
*CytB*	J	10,290–11,432	1095		ATG	TAG	3
*trnS2* (UCN)	J	11,431–11,495	65	TGA			−2
*ND1*	N	11,512–12,462	933		ATT	TAG	16
*trnL2* (CUN)	N	12,464–12,529	66	TAG			1
*lrRNA*	N	12,530–13,823	1358				0
*trnV*	N	13,824–13,893	70	TAC			0
*srRNA*	N	13,894–14,690	798				0
Control region		14,691-17,066	2376				0

* represents gene spacing, and negative numbers represent the number of overlapping nucleotides between adjacent genes.

## Data Availability

The data that support the findings of this study will be available in GenBank at https://www.ncbi.nlm.nih.gov/ (accessed on 29 July 2024), with accession number PP265270-PP265272.

## Supplementary Materials

The following supporting information can be downloaded at: https://www.mdpi.com/article/10.3390/genes15081022/s1, Figure S1: Phylogenetic relationships of Scarabaeidae based on 13 PCG dataset using maximum likelihood; Figure S2: Phylogenetic trees of Scarabaeidae inferred by the MrBayes 3.2.6 method based on 13 PCGs; Figure S3: Phylogenetic trees of Scarabaeidae inferred by the maximum likelihood method based on amino acid sequences of PCGs.
