# Supplementary material for: Elucidating Scarab Divergence in an Evolutionary-Ecological Context through the Comprehensive Analysis of the Complete Mitogenome of Anomala
Source: Genes (Basel). 2024 Aug 3;15(8):1022. doi: 10.3390/genes15081022 (PMC11353958; doi:10.3390/genes15081022)
Supplement: Supplementary file 1 [file genes-15-01022-s001.zip › genes-3107878-supplementary.pdf]

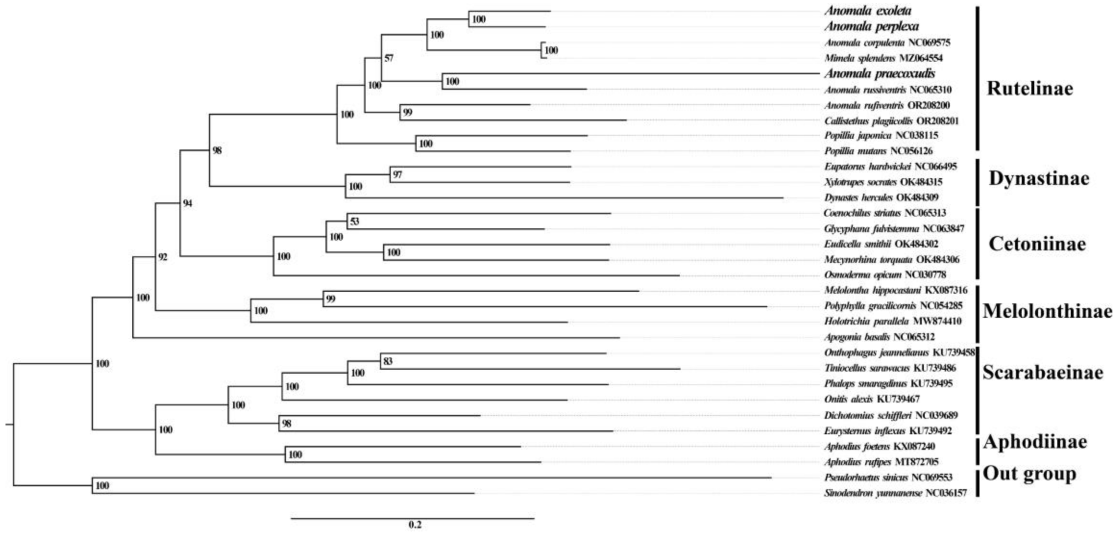

Figure S1 Phylogenetic trees of Scarabaeidae inferred by maximum likelihood methods based on sequences of PCGs

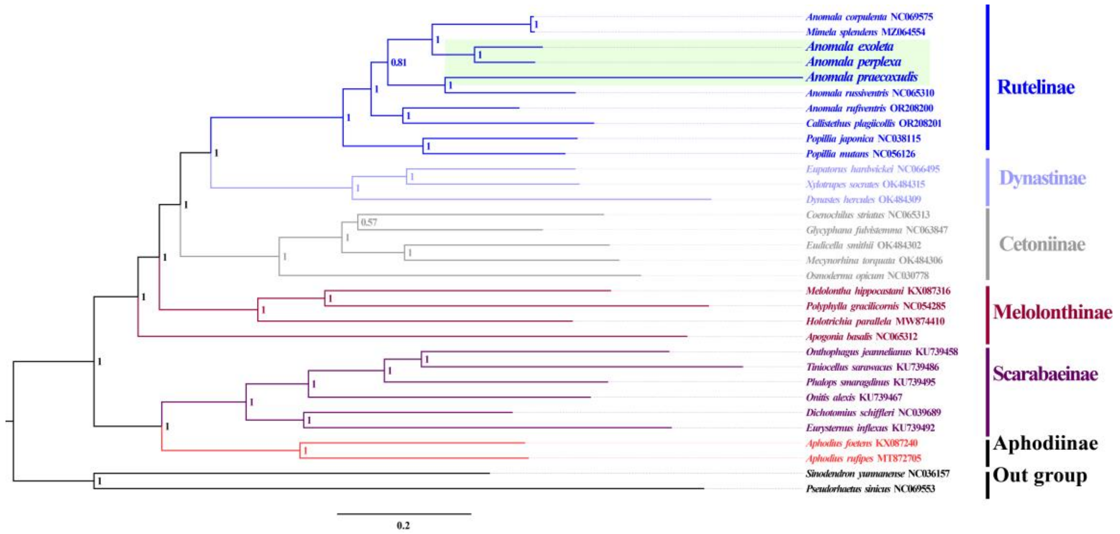

Figure S2 Phylogenetic trees of Scarabaeidae inferred by the MrBayes 3.2.6 methods based 13 PCGs

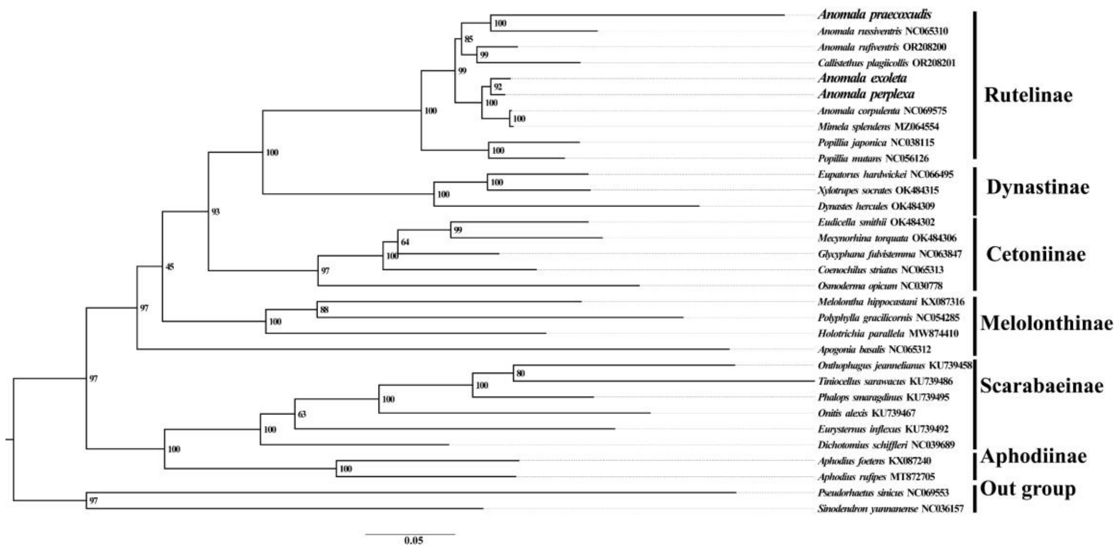

Figure S3 Phylogenetic trees of Scarabaeidae inferred by the inferred by maximum likelihood methods based amino acid of sequences of PCGs
